# One‐Year Outcomes of Topography‐Guided LASIK for Myopia and Astigmatism

**DOI:** 10.1155/joph/3410286

**Published:** 2026-01-30

**Authors:** Shanshan Wei, Yan Zheng, Caiyun Fu, Li Zhang, Yabin Hu, Yiran Dong, Dongyue Ma, Changbin Zhai

**Affiliations:** ^1^ Beijing Institute of Ophthalmology, Beijing Tongren Eye Center, Beijing Tongren Hospital, Beijing Ophthalmology & Visual Sciences Key Laboratory, Capital Medical University, Beijing, China, ccmu.edu.cn

**Keywords:** astigmatism, higher-order aberrations, myopia, personalized refractive surgery, topography-guided LASIK, visual quality

## Abstract

**Introduction:**

To assess the efficacy and safety of topography‐guided LASIK (TG‐LASIK) in correcting myopia and astigmatism and to evaluate clinical outcomes and visual quality postoperatively.

**Methods:**

We conducted a prospective study including patients aged 18–40 years with stable refraction for over 12 months. The outcomes measured were uncorrected distance visual acuity (UDVA), contrast sensitivity, higher‐order aberrations (HOAs), and patient‐reported visual quality using a validated Quality of Vision (QoV) questionnaire.

**Results:**

A total of 86 eyes of 43 patients were analyzed. At 12 months postoperatively, 97% of the eyes achieved a UDVA of 20/20 or better, and 99% maintained or improved their corrected distance visual acuity (CDVA). The mean UDVA remained stable over time (*p* > 0.05). The correction index for astigmatism was 0.99, with a strong correlation between target‐induced astigmatism (TIA) and surgically induced astigmatism (SIA) (*R* ^2^ = 0.9751). Contrast sensitivity improved significantly at all spatial frequencies postoperatively (*p* < 0.05) and remained stable after 6 months. The QoV questionnaire revealed that blurred vision and fluctuations in vision were the most frequently reported symptoms, with fluctuation being the most bothersome. HOAs and coma increased significantly after surgery (*p* < 0.05), whereas lower‐order aberrations (LOAs) and spherical aberration remained unchanged (*p* > 0.05).

**Conclusions:**

TG‐LASIK is an effective approach in personalized refractive surgery, demonstrating safety and efficacy in improving visual quality for myopia and astigmatism. The improvement in visual quality, despite an increase in HOAs, suggests the effectiveness of personalized ablation profiles.

## 1. Introduction

Femtosecond laser‐assisted in situ keratomileusis (FS‐LASIK) is one of the most popular refractive surgeries worldwide, and its safety and efficiency have been well established [1]. To improve patient satisfaction and visual quality, FS‐LASIK surgery has undergone significant advancements, from traditional laser ablation to customized ablation including the wavefront‐optimized laser‐assisted in situ keratomileusis (WFO‐LASIK), the wavefront‐guided laser‐assisted in situ keratomileusis (WFG‐LASIK), and the topography‐guided laser‐assisted in situ keratomileusis (TG‐LASIK) [2–6]. TG‐LASIK preserves the natural aspheric curvature of the cornea and corrects corneal irregularities [7]. Previous studies have concluded that TG‐LASIK surgery allows for more precise correction of refractive errors and reduces higher‐order aberrations (HOAs), ultimately improving optical quality beyond what glasses or contact lenses can achieve [8–10]. This study aimed to evaluate the postoperative correction outcomes of the TG‐LASIK.

## 2. Materials and Methods

### 2.1. Subjects and Dataset

This prospective study included patients from October 2021 to March 2023 at the Beijing Tongren Hospital. Inclusion criteria were age 18–40 years, stable refraction condition for more than 1 year, corrected distance visual acuity reaching 20/25, and manifest refraction consistent with myopia or myopic/mixed astigmatism, with cylinder ≤ −5.00 D. Patients with any ocular diseases or suspected keratoconus were excluded from this study. All included patients completed several preoperative assessments including uncorrected distance visual acuity (UDVA), manifest and cycloplegic refraction, noncontact intraocular pressure, corrected distance visual acuity (CDVA), slit‐lamp microscopy, dilated fundus examination, Topographic Modeling System (TMS‐4, Tomey Corporation, Nagoya, Aichi, Japan), reference biometer (Lenstar LS 900, HAAG‐STREIT AG, Switzerland), corneal topography (Vario Topolyzer, WaveLight, Alcon Laboratories, Inc., Fort Worth, TX, United States), and Pentacam Scheimpflug (Oculus Optikgerate GmbH, Wetzlar, Germany). Our study adhered to the tenets of the Declaration of Helsinki, and the study received ethics approval from the Beijing Tongren Hospital Medical Ethics Committee.

### 2.2. Surgical Planning and Technique

We used the WaveLight Topolyzer VARIO to obtain 4–8 high‐quality images for each eye to ensure the stability and reproducibility of the data. Corneal topography data were subjected to rigorous selection based on the following criteria: (1) the edges of the pupil and reflection ring must be correctly identified by the software. (2) There should be no obvious breaks in the reflection ring, and the image should not be significantly affected by shadows, such as those caused by the eyelids, eyelashes, nasal bridge, or dry tear film. (3) The data coverage area should exceed 70% of the analyzed area. Within the 6.5 mm diameter range, data coverage should exceed 90%. (4) A low “median absolute deviation” (MAD) score is required (< 0.10), indicating a high degree of consistency in the scan results. Images that did not meet the aforementioned criteria were excluded. If fewer than four acceptable images are obtained, the data‐acquisition process must be repeated. Finally, high‐quality data were imported into Contoura Vision (Alcon Vision, LLC, Fort Worth, TX, USA) topography‐guided ablation platform to generate personalized corneal ablation profiles. The treatment planning was based on the manifest refraction and the topography data obtained from the Topolyzer Vario, following the principles of the topography‐modified refraction (TMR) approach. The Phorcides Analytical Engine was not used.

All surgeries were performed by the same experienced surgeon using a WaveLight FS200 femtosecond laser (Alcon Vision, LLC, Fort Worth, TX, USA) for corneal flap creation. The flap thickness ranged from 100 to 110 μm, diameter of 8.5 mm, 90° side‐cut angle, and 50° hinge angle. Excimer ablation was performed using the WaveLight EX500 laser (Alcon Vision, LLC, Fort Worth, TX, USA), with an optical zone set at 6.5 mm.

### 2.3. Quality of Vision (QoV) Questionnaire

All patients were asked to complete a validated QoV questionnaire developed by McAlinden et al. [11, 12]. The questionnaire is a reliable and standardized tool for evaluating QoV after refractive correction or ophthalmic surgery [13, 14]. The 30‐item questionnaire included the following 10 symptoms: glare, halos, starbursts, hazy vision, blurred vision, distortion, double images, fluctuating vision, focusing difficulties, and difficulty in judging distance/depth perception. Patients were required to rate each symptom on a 4‐point scale with the frequency (never, 0; occasionally, 1; quite often, 2; and very often, 3), severity (not at all, 0; mild, 1; moderate, 2; and severe, 3), and bothersome (not at all, 0; a little, 1; quite, 2; and very, 3).

### 2.4. Wavefront Aberrations Measurement

Corneal wavefront aberrations were measured using Pentacam Scheimpflug (Oculus Optikgerate GmbH, Wetzlar, Germany) including the total corneal higher‐order aberration root mean square (HOA RMS), total lower‐order aberration root mean square (LOA RMS), spherical aberration (SA), and coma of the anterior corneal surface. The cornea was analyzed with a standardized diameter of 6 mm.

### 2.5. Contrast Sensitivity

Contrast sensitivity was measured using a Functional Vision Analyzer (Vision Tester, Stereo Optical Company Inc., Chicago, IL, United States) under mesopic condition. The spatial frequencies of the stimuli were 1.5, 3.0, 6.0, 12.0, and 18.0 cycles per degree (c/d).

### 2.6. Postoperative Assessment and Follow‐Up

The patients attended follow‐up appointments at 1 day, 1 week, 1 month, 3 months, 6 months, and 12 months after surgery. During each follow‐up, a refractive examination was performed. Additionally, preoperatively and at 1, 3, 6, and 12 months postoperatively, patients completed questionnaires and underwent Pentacam and contrast sensitivity evaluations.

### 2.7. Statistical Analysis

The Shapiro–Wilk test was used to assess data normality. For variables with a normal distribution, analysis was performed using the paired *t*‐test and repeated measures ANOVA. Nonnormally distributed data were analyzed using the Wilcoxon signed‐rank test and Friedman test. Statistical analyses were performed using SPSS Version 23.0, and GraphPad Prism 9 was used for graph generation. Astigmatism vector analysis was conducted at AstigMATIC (https://www.lasikbbs.com) based on the Alpins method. A *p* value less than 0.05 was considered statistically significant.

**FIGURE 1:**
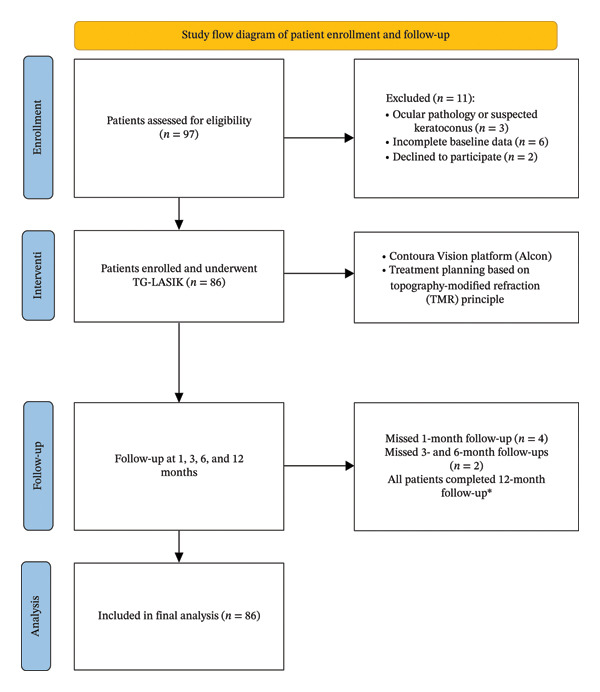
Study flow diagram of patient enrollment and follow‐up.

A flow diagram summarizing the patient enrollment and follow‐up process is presented in Figure 1.

## 3. Results

Our study included 86 eyes from 43 patients, consisting of 11 males (25.6%) and 32 females (74.4%), with an average age of 27.71 ± 4.63 years (ranging from 19 to 40 years). During the 12‐month follow‐up, 4 patients missed their 1‐month postoperative visit and another 2 missed their 3‐month and 6‐month follow‐ups. All patients attended the 12‐month follow‐up (Figure 1).

### 3.1. Preoperative Data

Table 1 shows the preoperative parameters of the included patients. All surgeries were completed without any complications.

**TABLE 1:** The preoperative parameters of the included patients.

Parameter	Value
UDVA (logMAR)	1.23 ± 0.32 (0.10, 2.0)
CDVA (logMAR)	−0.04 ± 0.05 (−0.08, 0.10)
Sphere (D)	−5.24 ± 2.28 (−9.75, 3.75)
Cylinder (D)	−2.09 ± 1.20 (−6.50, 0)
SE (D)	−6.29 ± 2.29 (−11.38, 0.50)
*K*_1_ (D)	42.65 ± 1.61 (36.62, 46.27)
*K*_2_ (D)	45.02 ± 1.76 (39.61, 49.10)
*K*_*m*_ (D)	43.82 ± 1.60 (39.22, 47.68)
SRI	0.19 ± 0.17 (0.01, 0.69)
SAI	0.35 ± 0.14 (0.17, 0.78)
Corneal thickness (μm)	541.73 ± 26.96 (482, 600)
Pupil diameter (mm)	4.51 ± 1.13 (2.87, 8.02)

*Note:* *K*_1_ = flat keratometry; *K*_2_ = steep keratometry; *K*_*m*_ = mean keratometry.

Abbreviations: CDVA, corrected distance visual acuity; SAI, surface asymmetry index; SE, spherical equivalent; SRI, surface regularity index; UDVA, uncorrected distance visual acuity.

### 3.2. Visual and Refractive Outcomes

**FIGURE 3:**
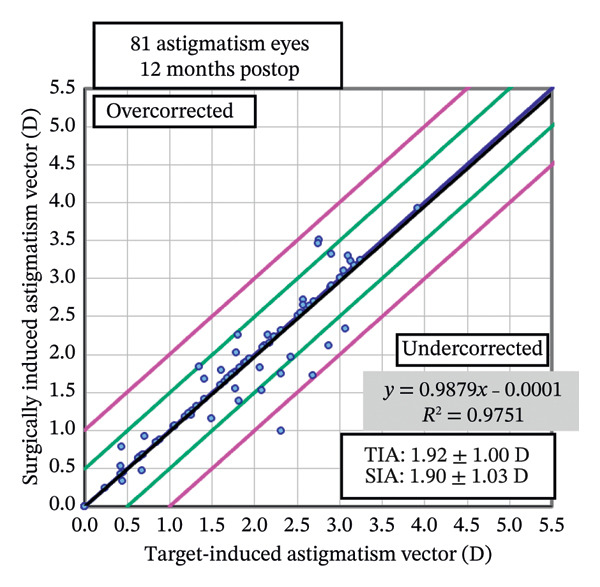
Scatter plot of target‐induced astigmatism (TIA) versus surgically induced astigmatism (SIA).

Postoperative visual acuity and refractive correction results are detailed in Table 2 and Figures 1 and 2, respectively. The mean UDVA improved slightly over time, with values of −0.07 ± 0.12 at 1 month, −0.08 ± 0.07 at 6 months, and −0.08 ± 0.08 at 12 months. There are no statistically significant differences in UDVA between the three time points, indicating stable visual acuity during the follow‐up period. The mean sphere showed significant differences across the follow‐up periods (*p* < 0.05). The mean cylinder remained stable, and no significant differences were observed. The mean SE was 0.06 ± 0.55 D at 1 month, −0.07 ± 0.44 D at 6 months, and −0.30 ± 0.58 D at 12 months, with significant differences observed between 1 and 12 months (*p* < 0.05) and between 6 and 12 months (*p* < 0.05). At 12 months after the surgery, 97% of the eyes achieved a UDVA of 20/20 or better (Figure 2(a)). A total of 99% of the eyes demonstrated an unchanged or better CDVA at 12 months postoperatively (Figure 2(b)). A scatterplot of the attempted versus achieved SE correction is shown in Figure 2(c). A total of 68% and 94% of the included eyes achieved an SE of ±0.50 D and ±1.00 D (Figure 2(d)). A total of 79% and 97% of the treated eyes had refractive astigmatism ≤ 0.50 D and ≤ 1.00 D (Figure 2(e)). From 3 months to 12 months after the surgery, 13% of the eyes had a change of more than 0.5 D in SE (Figure 2(f)). Standard plots for vector analysis derived using the Alpins method are shown in Figure 3. The target‐induced astigmatism (TIA) versus surgically induced astigmatism (SIA) scatter plot revealed a strong linear correlation (*R* ^2^ = 0.9751), with most points within ±0.50 D of the identity line. The mean CI was 0.99.

**TABLE 2:** The postoperative visual acuity and refractive correction results.

Parameter	1M postop	6M postop	12M postop	*p* value	*p* value 1M–6M	*p* value 1M–12M	*p* value 6M–12M
*UDVA* (*logMAR*)							
Mean ± SD	−0.07 ± 0.12	−0.08 ± 0.07	−0.08 ± 0.08	< 0.05	0.076	0.099	0.899
Range	−0.18, 0.7	−0.18, 0.05	−0.18, 0.4				

*Sphere* (*D*)							
Mean ± SD	0.17 ± 0.58	0.08 ± 0.50	−0.21 ± 0.62	< 0.05	0.218	< 0.05	< 0.05
Range	−2.0, 1.87	−1.0, 1.25	−2.0, 1.5				

*Cylinder* (*D*)							
Mean ± SD	−0.21 ± 0.40	−0.30 ± 0.35	−0.20 ± 0.34	0.244			
Range	−1.25, 0.88	−1.25, 0	−1.5, 0.62				

*SE* (*D*)							
Mean ± SD	0.06 ± 0.55	−0.07 ± 0.44	−0.30 ± 0.58	< 0.05	0.085	< 0.05	< 0.05
Range	−2.25, 1.44	−1.0, 1.0	−2.25, 1.13				

Abbreviations: SE, spherical equivalent; UDVA, uncorrected distance visual acuity.

FIGURE 2The postoperative visual acuity and refractive correction results. (a) Uncorrected distance visual acuity. (b) Change in corrected distance acuity. (c) Spherical equivalent attempted vs. achieved. (d) Spherical equivalent refractive accuracy. (e) Refractive astigmatism. (f) Stability of spherical equivalent refraction.

**Figure:**
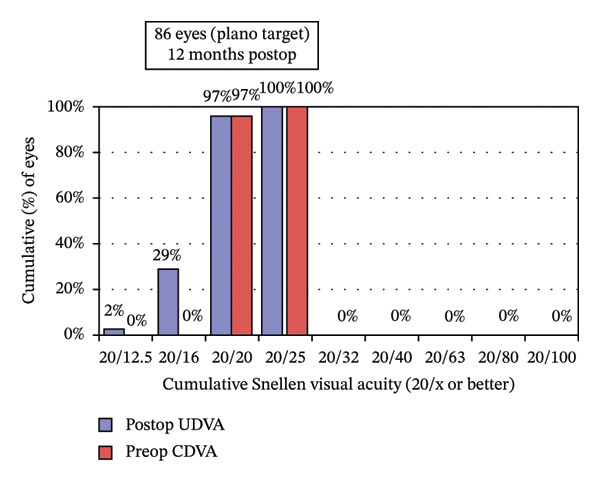
(a)

**Figure:**
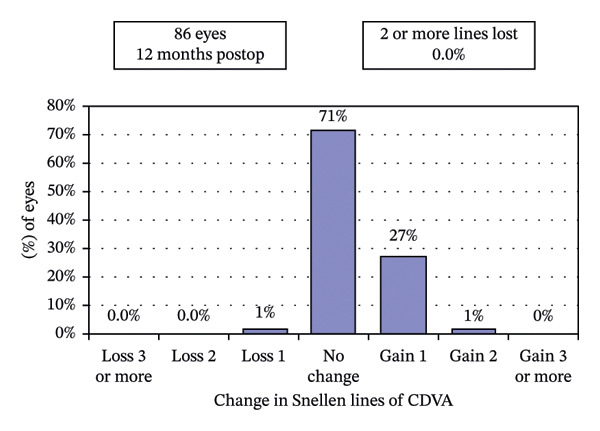
(b)

**Figure:**
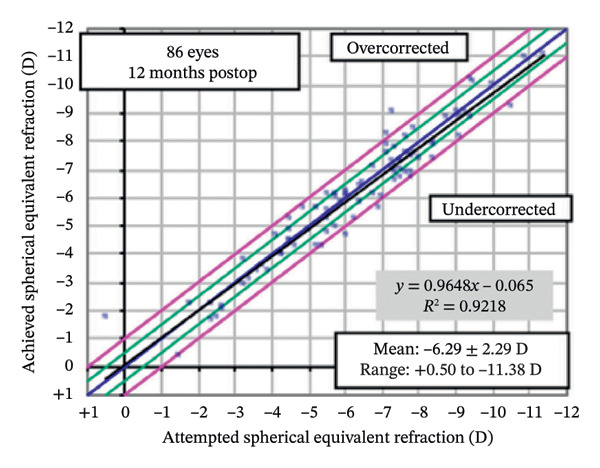
(c)

**Figure:**
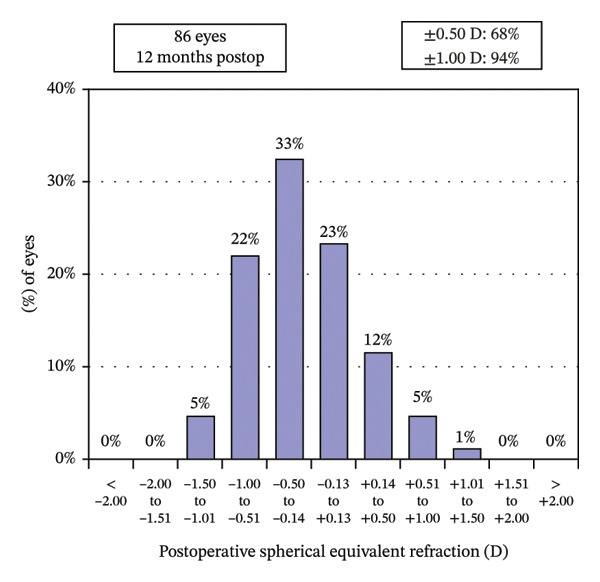
(d)

**Figure:**
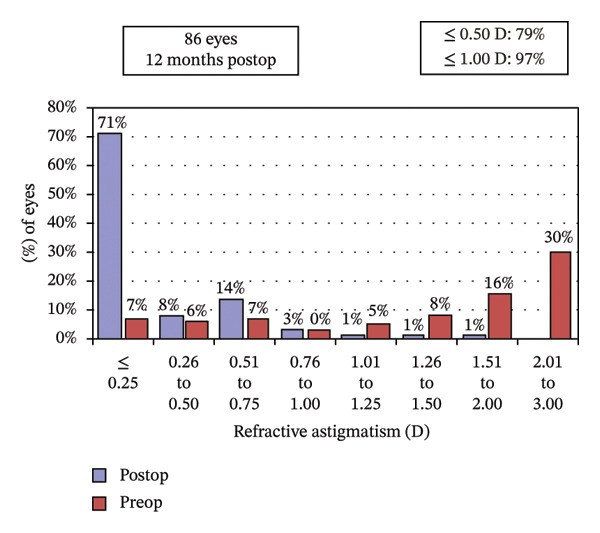
(e)

**Figure:**
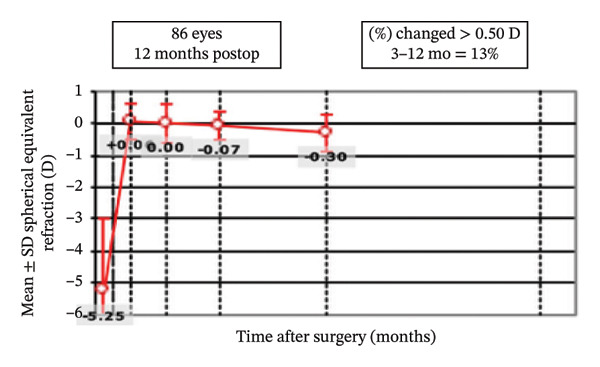
(f)

### 3.3. QoV Scores

**FIGURE 4:**
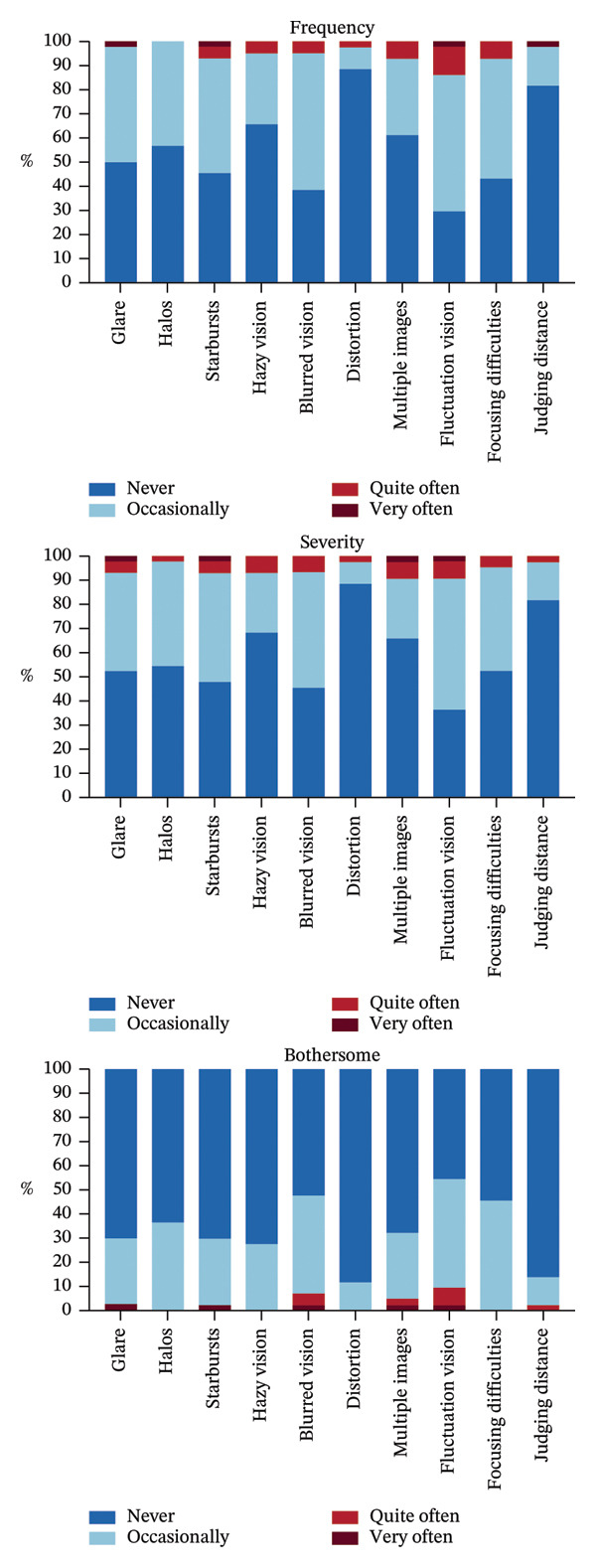
Stacked histogram representing the frequency, severity, and bothersome of each symptom 12 months after TG‐LASIK.

Table 3 presents the mean QoV scores for each symptom, rated from 0 to 3 based on frequency, severity, and bothersome. Blurred vision and fluctuation vision were reported most frequently at 12 months after surgery (Table 3). Figure 4 summarizes the detailed outcomes of all 10 symptoms in the TG‐LASIK group 12 months postoperatively. In the frequency analysis, fluctuation vision had the highest number of “quite often” and “very often” ratings, shown in red and dark red. In the severity analysis, multiple images and fluctuation vision had the most “quite often” and “very often” ratings. Similarly, in the bothersome analysis, fluctuation vision also had the highest number of “quite often” and “very often” ratings. The detailed scores for each QoV symptom are provided in Table S1.

**TABLE 3:** The mean QoV scores for each symptom.

Symptom	Frequency	Severity	Bothersome
Glare	0.55 ± 0.63	0.57 ± 0.70	0.34 ± 0.61
Haloes	0.43 ± 0.50	0.48 ± 0.55	0.36 ± 0.49
Starbursts	0.64 ± 0.69	0.61 ± 0.69	0.34 ± 0.61
Hazy vision	0.39 ± 0.58	0.39 ± 0.62	0.27 ± 0.45
Blurred vision	0.66 ± 0.57	0.61 ± 0.62	0.57 ± 0.70
Distortion	0.14 ± 0.41	0.14 ± 0.41	0.11 ± 0.32
Multiple images	0.45 ± 0.63	0.45 ± 0.73	0.39 ± 0.65
Fluctuation vision	0.86 ± 0.70	0.75 ± 0.69	0.66 ± 0.71
Focusing difficulties	0.64 ± 0.61	0.52 ± 0.59	0.45 ± 0.50
Judging Distance	0.20 ± 0.46	0.20 ± 0.46	0.16 ± 0.43

The most frequently reported symptom 12 months after the surgery was fluctuation vision, occurring quite often or very often in 14.0% of patients, moderate or more severe in 9.3%, and considered quite often or very often bothersome by 9.3%. It is indicated that the frequency and severity scores were comparable, whereas bothersome scores remained noticeably lower.

### 3.4. Wavefront Aberrations Measurement

The wavefront aberrations data, including HOA RMS, LOA RMS of corneal, corneal SA, and coma before and 12 months after TG‐LASIK are shown in Table 4. At 12 months postoperatively, RMS HOA and coma increased significantly (*p* < 0.05). There were no significant changes in the RMS LOA and SA.

**TABLE 4:** The data of wavefront aberrations before and 12 months after TG‐LASIK.

	**Pre**	**12M postop**	**T**	**p**

HOA RMS	0.42 ± 0.11	0.84 ± 0.30	12.061	< 0.05
LOA RMS	2.37 ± 0.78	2.25 ± 0.76	−1.04	0.543
SA	0.27 ± 0.19	0.27 ± 0.19	2.66	0.330
Coma	0.37 ± 0.28	0.50 ± 0.29	0.39	< 0.05

Abbreviations: HOA RMS, higher‐order aberrations root mean square; LOA RMS, lower‐order aberrations root mean square; SA, spherical aberration.

### 3.5. Contrast Sensitivity

**FIGURE 5:**
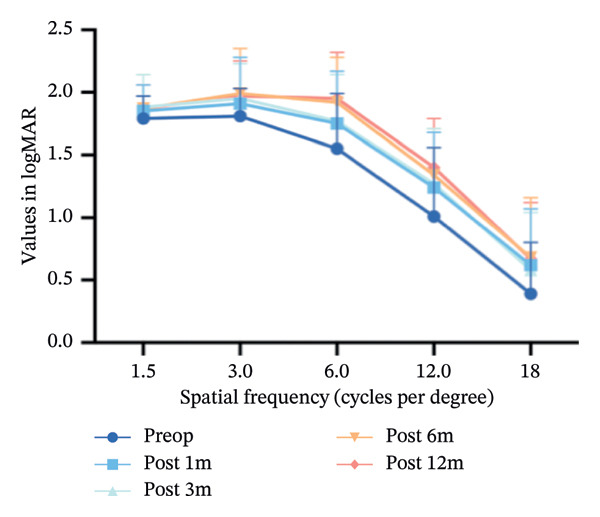
The changes in spatial frequency before and after the surgery.

Figure 5 illustrates the changes in spatial frequency before and after surgery. Preoperatively, the spatial frequency was lower than that of the postoperative measurements at all follow‐up times. After 6 months, the contrast sensitivity remained relatively stable.

## 4. Discussion

Our research focused on evaluating the outcomes of TG‐LASIK surgery. We propose that TG‐LASIK may offer superior correction for refractive errors and improve postoperative visual quality.

In our study, we observed that visual acuity and quality 12 months after TG‐LASIK surgery were associated with high levels of accuracy and satisfaction. None of the patients experienced a loss of two or more lines of vision, and the scatter plot along with the linear regression analysis indicated that the surgery was capable of precisely achieving the intended refractive state. While cylinder remained stable throughout follow‐up, both sphere and SE showed statistically significant changes between 1 and 12 months and between 6 and 12 months, indicating a mild trend toward myopic regression. This regression may be attributed to factors such as corneal epithelial remodeling and biomechanical changes, as similarly suggested by previous studies [15]. Similar outcomes have been linked to central epithelial thickness increases of ∼3–5 μm postoperatively, which may induce minor refractive shifts [16, 17]. Importantly, these changes did not affect final outcomes, as 97% of eyes achieved 20/20 or better UDVA at 12 months. These findings, combined with improved scores in multiple QoV dimensions, suggest that patients subjectively perceived better visual quality after surgery. Furthermore, vector analysis of astigmatic correction demonstrated excellent alignment between intended and achieved astigmatic targets, with CI close to 1.00 and minimal axis deviation. Overall, these results suggest that TG‐LASIK surgery is safe, effective, and accurate for the correction of refractive errors, which is consistent with the results of previous research studies [10, 18–24].

We conducted QoV to assess patient satisfaction and subjective vision quality following TG‐LASIK surgery. The results indicated that patients who underwent TG‐LASIK demonstrated improvements in various visual functions at the 12‐month postoperative mark, albeit with an increase in the frequency, severity, and bothersome of visual fluctuations. This could be attributed to factors such as tear film stability and potential ocular surface irregularities. On the other hand, there was a significant enhancement in judging distance, with improvements noted in both frequency and bothersome, suggesting an enhancement in patients’ spatial awareness. Previous studies have reported patient satisfaction with TG‐LASIK using the PROWL survey [25]. Compared with our 30‐item QoV, the PROWL questionnaire encompassed similar symptoms such as multiple images, glares, halos, and starbursts, and it is demonstrated that TG‐LASIK significantly improved these symptoms. In contrast, our study showed that there was no significant change in these symptoms postoperatively, except for multiple images, which showed improvement. A possible explanation for this discrepancy could be that our study had a longer follow‐up period, and the timing of symptom assessments might have affected the results. Moreover, our evaluation used a multidimensional approach to assess symptoms by frequency, severity, and bothersome, which was different from that of a previous study. Compared with a study analyzing the subjective quality of vision after SMILE using the same QoV questionnaire, glare, halos, and starbursts were the most commonly reported QoV symptoms. However, in our study, the scores for these three symptoms were lower than those reported for SMILE. Conversely, blurred vision, distortion, multiple images, and fluctuation vision showed higher scores in our analysis than in the SMILE studies [26]. We speculate that glare, halos, and starbursts may be mitigated by performing topography‐guided treatment. However, given the lack of a direct comparison, these findings should be interpreted cautiously. Further investigations are needed to clarify the differences.

Compared with traditional visual acuity examinations, contrast sensitivity provides a more comprehensive assessment of overall visual system quality and function [26, 27]. Our results demonstrated an enhancement in contrast sensitivity postoperatively at all spatial frequencies, which was in accordance with recent studies that indicated an improvement in contrast sensitivity postoperatively, despite an increase in HOAs [28, 29]. This may be explained by improved corneal regularity, neural adaptation, or selective aberration profiles such as mild spherical aberration improving depth of focus. Moreover, the TG‐LASIK procedure appears to have played a role in improving contrast sensitivity by surgically inducing fewer HOAs [30, 31]. This aligns with previous reports suggesting that TG‐LASIK may induce fewer HOAs compared with SMILE and wavefront‐optimized LASIK, potentially due to its customized ablation pattern guided by anterior corneal topography [7, 32].

Our analysis revealed a statistically significant increase in HOAs and coma, which is consistent with many previous studies [10, 22, 33]. Despite this increase, visual quality and contrast sensitivity either remained stable or even improved postoperatively. There is substantial evidence that HOAs alone are insufficient to fully account for visual quality and may have been partially mitigated by factors such as improved corneal regularity and neural adaptation. The TG‐LASIK might also have contributed to minimizing the functional impact of induced aberrations [25, 34–36]. Kim’s study demonstrated a significant increase in postoperative HOAs and coma, which is consistent with our study. However, our results showed no significant change in SA postoperatively, in contrast to Kim’s report of a significant increase in SA [10]. This discrepancy may cause of study populations or preoperative corneal characteristics. As we did not further explore the contributing factors in this study, future investigations are needed to clarify the cause. There are some studies have reported a reduction in HOAs following TG‐LASIK, which appears to contradict our findings. Upon closer examination, these discrepancies may be explained by differences in study populations and preoperative corneal conditions. For instance, HOA reduction is more likely in eyes with highly irregular corneas, such as those with early keratoconus or highly irregular preoperative corneal profiles [37]. In these cases, the goal is not purely refractive correction but also corneal regularization, which can indeed reduce HOAs.

In addition, our inclusion criteria followed the TMR principle. This inclusion threshold was chosen to better represent real‐world TG‐LASIK practice and to enable individualized, cornea‐based ablation planning. Although a wider range may introduce slight variability in residual astigmatism, it more accurately reflects clinical populations encountered in routine refractive surgery.

Although our study provides valuable insights into the application and outcomes of TG‐LASIK, several limitations must be acknowledged. First, our analysis was based on a single‐center experience, which may not capture the full spectrum of patient diversity and surgical outcomes that a multicenter study might reveal. The relatively small sample size of our study could introduce bias and limit the generalizability of our findings to broader patient populations with varying degrees of refractive error and corneal conditions. Additionally, while our study benefits from a prospective design, providing direct observations of the outcomes of TG‐LASIK, it is not without its limitations. One such limitation is the lack of detailed subgroup analysis based on the refractive status of patients. For instance, we did not specifically examine postoperative outcomes in patients with different levels of astigmatism, which may obscure treatment effects and potential predictive factors within specific refractive subgroups. Future studies should consider a more nuanced stratification of patients based on refractive conditions to more accurately assess the impact of different refractive characteristics on surgical outcome. Moreover, our study did not include a direct comparison with other surgical methods, such as wavefront‐guided LASIK or SMILE surgery. Such comparisons are essential for a comprehensive assessment of the relative advantages and limitations of topography‐guided LASIK among the refractive surgery techniques. Future studies should incorporate comparative analyses of various surgical methods.

In summary, this study demonstrates that TG‐LASIK achieves stable refractive and visual outcomes over 1 year, providing a reliable foundation for long‐term visual quality improvement.

## 5. Conclusion

In conclusion, our prospective, single‐center study provides one of the most comprehensive 1‐year evaluations of TG‐LASIK outcomes to date, integrating refractive accuracy, HOAs, contrast sensitivity, and subjective visual quality. Using a 30‐item multidimensional QoV questionnaire, we demonstrated consistent improvement in both objective and subjective visual parameters, confirming the long‐term stability and visual quality benefits of TG‐LASIK based on the TMR principle in East Asian eyes. In the future, we will further compare TG‐LASIK with other personalized refractive techniques to advance individualized vision correction.

## Funding

This work was supported by the National Key Research and Development Program of China (No. 2022YFC2404505) and the Wu Jieping Medical Foundation (Grant No. 320.6750).

## Conflicts of Interest

The authors declare no conflicts of interest.

## Supporting Information

Table S1. Quality of Vision (QoV) scores assessed preoperatively and at 12 months postoperatively, including three dimensions: frequency, severity, and bothersomeness.

## Supporting information


**Supporting Information** Additional supporting information can be found online in the Supporting Information section.

## Data Availability

The datasets used in the study are available from the corresponding author upon reasonable request.
