# Supplementary material for: One‐Year Outcomes of Topography‐Guided LASIK for Myopia and Astigmatism
Source: J Ophthalmol. 2026 Jan 30;2026:3410286. doi: 10.1155/joph/3410286 (PMC12857243; doi:10.1155/joph/3410286)
Supplement: Supplementary file 1 — Supporting Information Additional supporting information can be found online in the Supporting Information section. [file JOPH-2026-3410286-s001.docx]

**Title page**

**Title:** One-Year Outcomes of Topography-Guided LASIK for Myopia and Astigmatism

**Each author's full name:** Shanshan Wei1, Yan Zheng1, Caiyun Fu1, Li Zhang1, Yabin Hu1, Yiran Dong, Dongyue Ma1, Changbin Zhai1.

**Institutional affiliation of each author:** 1 Beijing Institute of Ophthalmology, Beijing Tongren Eye Center, Beijing Tongren Hospital, Capital Medical University, Beijing Ophthalmology & Visual Sciences Key Laboratory, Beijing, China.

**Corresponding author**: Changbin Zhai

Beijing Institute of Ophthalmology, Beijing Tongren Eye Center, Beijing Tongren Hospital, Capital Medical University, Beijing Ophthalmology & Visual Sciences Key Laboratory, Beijing, China.

Email: [eyedrzcb@163.com](mailto:eyedrzcb@163.com)

Phone number:+86 15810911180

**Table S1.** The detailed scores for each QoV symptom

Frequency

| Parameter | Glare | Halo | Starbursts | Hazy vision | Blurred vision | Distortion | Multiple  images | Fluctuation vision | Focusing difficulties | Judging distance |
| --- | --- | --- | --- | --- | --- | --- | --- | --- | --- | --- |
| Preoperative | | | | | | | | | | |
| Mean | 0.47 | 0.45 | 0.67 | 0.36 | 0.93 | 0.05 | 0.64 | 0.67 | 0.52 | 0.43 |
| Standard deviation | 0.74 | 0.63 | 0.79 | 0.62 | 1.02 | 0.22 | 0.82 | 0.53 | 0.59 | 0.63 |
| Minium | 0 | 0 | 0 | 0 | 0 | 0 | 0 | 0 | 0 | 0 |
| Maximum | 3 | 3 | 3 | 3 | 3 | 1 | 3 | 2 | 2 | 2 |
| Never (0) | 27 | 25 | 21 | 29 | 18 | 40 | 22 | 15 | 22 | 27 |
| Occasionally (1) | 11 | 14 | 15 | 12 | 14 | 2 | 14 | 26 | 18 | 12 |
| Quite often (2) | 3 | 0 | 5 | 0 | 5 | 0 | 2 | 1 | 2 | 3 |
| Very often (3) | 1 | 1 | 1 | 1 | 5 | 0 | 2 | 0 | 0 | 0 |
| 12 months postoperative | | | | | | | | | | |
| Mean | 0.53 | 0.44 | 0.63 | 0.40 | 0.65 | 0.14 | 0.46 | 0.88 | 0.63 | 0.18 |
| Standard deviation | 0.63 | 0.50 | 0.69 | 0.58 | 0.57 | 0.41 | 0.63 | 0.70 | 0.62 | 0.45 |
| Minium | 0 | 0 | 0 | 0 | 0 | 0 | 0 | 0 | 0 | 0 |
| Maximum | 3 | 1 | 3 | 2 | 2 | 2 | 2 | 9 | 2 | 2 |
| Never (0) | 22 | 24 | 20 | 28 | 16 | 38 | 26 | 12 | 18 | 35 |
| Occasionally (1) | 20 | 19 | 20 | 13 | 25 | 4 | 14 | 25 | 22 | 7 |
| Quite often (2) | 0 | 0 | 2 | 2 | 2 | 1 | 3 | 5 | 3 | 1 |
| Very often (3) | 1 | 0 | 1 | 0 | 0 | 0 | 0 | 1 | 0 | 0 |
| *P* | 0.696 | 1 | 0.638 | 0.658 | 0.137 | 0.414 | 0.168 | *P*<0.05 | 0.244 | *P*<0.05 |

Severity

| Parameter | Glare | Halo | Starbursts | Hazy Vision | Blurred Vision | Distortion | Multiple  images | Fluctuation vision | Focusing Difficulties | Judging Distance |
| --- | --- | --- | --- | --- | --- | --- | --- | --- | --- | --- |
| Preoperative | | | | | | | | | | |
| Mean | 0.50 | 0.50 | 0.60 | 0.36 | 0.93 | 0.05 | 0.64 | 0.60 | 0.55 | 0.33 |
| Standard deviation | 0.71 | 0.67 | 0.73 | 0.66 | 1.07 | 0.22 | 0.82 | 0.59 | 0.67 | 0.53 |
| Minium | 0 | 0 | 0 | 0 | 0 | 0 | 0 | 0 | 0 | 0 |
| Maximum | 2 | 2 | 2 | 3 | 3 | 1 | 3 | 2 | 2 | 2 |
| Never (0) | 26 | 25 | 23 | 30 | 20 | 40 | 22 | 19 | 23 | 29 |
| Occasionally (1) | 11 | 11 | 13 | 10 | 10 | 2 | 14 | 21 | 15 | 12 |
| Quite often (2) | 5 | 4 | 6 | 1 | 7 | 0 | 2 | 2 | 4 | 1 |
| Very often (3) | 0 | 0 | 0 | 1 | 5 | 0 | 2 | 0 | 0 | 0 |
| 12 months postoperative | | | | | | | | | | |
| Mean | 0.56 | 0.49 | 0.60 | 0.40 | 0.60 | 0.14 | 0.47 | 0.77 | 0.51 | 0.19 |
| Standard deviation | 0.70 | 0.55 | 0.69 | 0.62 | 0.52 | 0.41 | 0.63 | 0.68 | 0.59 | 0.45 |
| Minium | 0 | 0 | 0 | 0 | 0 | 0 | 0 | 0 | 0 | 0 |
| Maximum | 3 | 2 | 3 | 2 | 2 | 2 | 2 | 3 | 2 | 2 |
| Never (0) | 23 | 23 | 21 | 29 | 19 | 38 | 28 | 15 | 22 | 35 |
| Occasionally (1) | 17 | 19 | 19 | 11 | 21 | 4 | 11 | 24 | 19 | 7 |
| Quite often (2) | 2 | 1 | 2 | 3 | 3 | 1 | 3 | 3 | 2 | 1 |
| Very often (3) | 1 | 0 | 1 | 0 | 0 | 0 | 1 | 1 | 0 | 0 |
| *P* | 0.661 | 1 | 0.890 | 0.701 | 0.072 | 0.414 | 0.142 | 0.101 | 0.833 | 0.109 |

Bothersome

| Parameter | Glare | Halo | Starbursts | Hazy Vision | Blurred Vision | Distortion | Multiple  images | Fluctuation vision | Focusing Difficulties | Judging Distance |
| --- | --- | --- | --- | --- | --- | --- | --- | --- | --- | --- |
| Preoperative | | | | | | | | | | |
| Mean | 0.38 | 0.36 | 0.36 | 0.33 | 0.76 | 0.02 | 0.64 | 0.55 | 0.45 | 0.31 |
| Standard deviation | 0.54 | 0.53 | 0.62 | 0.61 | 1.01 | 0.15 | 0.88 | 0.63 | 0.55 | 0.47 |
| Minium | 0 | 0 | 0 | 0 | 0 | 0 | 0 | 0 | 0 | 0 |
| Maximum | 2 | 2 | 3 | 3 | 3 | 1 | 3 | 2 | 2 | 1 |
| Never (0) | 27 | 28 | 22 | 30 | 23 | 41 | 23 | 22 | 24 | 29 |
| Occasionally (1) | 14 | 13 | 14 | 11 | 10 | 1 | 13 | 17 | 17 | 13 |
| Quite often (2) | 1 | 1 | 4 | 0 | 5 | 0 | 1 | 3 | 1 | 0 |
| Very often (3) | 0 | 0 | 0 | 1 | 4 | 0 | 3 | 0 | 0 | 0 |
| 12 months postoperative | | | | | | | | | | |
| Mean | 0.35 | 0.37 | 0.35 | 0.28 | 0.58 | 0.11 | 0.40 | 0.67 | 0.44 | 0.14 |
| Standard deviation | 0.61 | 0.49 | 0.61 | 0.45 | 0.70 | 0.32 | 0.66 | 0.71 | 0.50 | 0.41 |
| Minium | 0 | 0 | 0 | 0 | 0 | 0 | 0 | 0 | 0 | 0 |
| Maximum | 3 | 1 | 3 | 1 | 3 | 1 | 3 | 3 | 1 | 2 |
| Never (0) | 30 | 27 | 30 | 31 | 22 | 38 | 29 | 19 | 23 | 37 |
| Occasionally (1) | 12 | 16 | 12 | 12 | 18 | 5 | 12 | 20 | 20 | 5 |
| Quite often (2) | 0 | 0 | 0 | 0 | 2 | 0 | 1 | 3 | 0 | 1 |
| Very often (3) | 1 | 0 | 1 | 0 | 1 | 0 | 1 | 1 | 0 | 0 |
| *P* | 0.694 | 0.782 | 0.054 | 0.617 | 0.275 | 0.180 | 0.060 | 0.175 | 1 | 0.071 |
